# Bacterial Community Structure and Predicted Function in Wheat Soil From the North China Plain Are Closely Linked With Soil and Plant Characteristics After Seven Years of Irrigation and Nitrogen Application

**DOI:** 10.3389/fmicb.2020.00506

**Published:** 2020-03-31

**Authors:** Geng Ma, Juan Kang, Jiarui Wang, Yulu Chen, Hongfang Lu, Lifang Wang, Chenyang Wang, Yingxin Xie, Dongyun Ma, Guozhang Kang

**Affiliations:** ^1^College of Agronomy, Henan Agricultural University, Zhengzhou, China; ^2^State Key Laboratory of Wheat and Maize Crop Science, Henan Agricultural University, Zhengzhou, China; ^3^National Engineering Research Centre for Wheat, Henan Agricultural University, Zhengzhou, China

**Keywords:** wheat, irrigation, nitrogen application, bacterial structure and function, Illumina MiSeq, PICRUSt

## Abstract

The influence of water and nitrogen (N) management on wheat have been investigated, but studies on the impact of long-term interactive water and N management on microbial structure and function are limited. Soil chemical properties and plants determine the soil microbial communities whose functions involved in nutrient cycling may affect plant productivity. There is an urgent need to elucidate the underlying mechanisms to optimize these microbial communities for agricultural sustainability in the winter wheat production area of the North China Plain. We performed high-throughput sequencing and quantitative PCR of the 16S rRNA gene on soil from a 7-year-old stationary field experiment to investigate the response of bacterial communities and function to water and N management. It was observed that water and N management significantly influenced wheat growth, soil properties and bacterial diversity. N application caused a significant decrease in the number of operational taxonomic units (OTUs), and both Richness and Shannon diversity indices, in the absence of irrigation. Irrigation led to an increase in the relative abundance of Planctomycetes, Latescibacteria, Anaerolineae, and Chloroflexia. In addition, most bacterial taxa were correlated with soil and plant properties. Some functions related to carbohydrate transport, transcription, inorganic ion transport and lipid transport were enriched in irrigation treatment, while N enriched predicted functions related to amino acid transport and metabolism, signal transduction, and cell wall/membrane/envelope biogenesis. Understanding the impact of N application and irrigation on the structure and function of soil bacteria is important for developing strategies for sustainable wheat production. Therefore, concurrent irrigation and N application may improve wheat yield and help to maintain those ecosystem functions that are driven by the soil microbial community.

## Introduction

Water and nitrogen (N) are the two main factors limiting crop productivity worldwide (Sinclair and Rufty, 2012). However, extensive use of chemical fertilizers has resulted in the degradation of soil physiochemical properties. The North China Plain is one of the most important wheat production areas in China (National Bureau of Statistics [NBS], 2013), and irrigation and N application are commonly used for winter wheat production in this area (Zhang et al., 2013; Wang C. et al., 2014). Most previous studies have focused on the influence of this pair of factors on ecosystem functions and higher level organisms (Qi et al., 2010; Lenka et al., 2013; Liu et al., 2018). However, these studies explored in-season effects of optimal water and N fertilizer management on crops, but not the sustainability of soil production capacity. Soil microbial biomass are the most abundant and ecologically important groups of organisms, and impact crop yield, soil productivity, and key ecosystem processes (Fierer et al., 2009; Marinari et al., 2010; Zhou et al., 2012; Agegnehu et al., 2016). However, their response to water and N, particularly in wheat field experiments, has not been examined.

Bacteria are the most abundant and diverse group of soil microorganisms, and influence soil function by driving biogeochemical processes, governing nutrient and organic matter composition as well as environmental functions (Jackson et al., 2003; Balser and Firestone, 2005). Understanding shifts in soil bacterial community structures in response to implementation of different agronomic approaches is important for improving soil fertility and function via specific management practices. Both water and N have a large influence on soil microbial communities, albeit through different mechanisms (Berg and Smalla, 2009). Water may relieve limitations related to soil water, stimulate microbial growth, and promote microbe and solute movement, thereby improving competitive intensity (Zhou et al., 2002). Water may also elevate soil pH and function through other mechanisms (Li et al., 2016). A previous study showed that long-term application of chemical N increased fungal abundance, while decreasing bacterial abundance in northeast China (Wang et al., 2019). N application increases inorganic labile soil N (NH_4_^+^-N and NO_3_^–^-N) content, which is advantageous for some soil microbes. However, N application may be unfavorable to soil microorganisms because it can decrease soil pH, and this is the most important ecological factor driving bacterial community structure (Fierer and Jackson, 2006; Rousk et al., 2010). Kavamura et al. (2018) observed that inorganic N affected the bacterial predicted function in wheat soil. Thus, understanding the effects of irrigation and N application on soil microbial communities is a priority, as is elucidating the underlying mechanisms driving winter wheat productivity in the North China Plain.

Although the influence on soil microbial community structure of agricultural management practices has received some attention over the past several decades, most studies have been conducted over a relatively short time, and the responses of the entire soil microbial community are poorly described (Edenborn and Sexstone, 2007; Martiny et al., 2011; Zhao S. et al., 2014). There appear to be few reports characterizing soil bacterial diversity, composition, and potential function through high-throughput sequencing following long-term irrigation and N application, understanding the relationships between bacteria and plants is an important step toward developing strategies for production of crops in a sustainable way.

We performed a stationary water and fertilization field experiment in 2010 using winter wheat, in which bacterial communities were analyzed by 16S rRNA marker sequencing (Hamady et al., 2008). The objective of the present study was to evaluate the effects of irrigation and N application on (1) wheat growth and soil chemical properties, (2) changes in the composition, abundance, and richness of soil bacterial communities, and (3) the potential function of soil bacteria. Finally, we aimed to determine the mechanisms by which irrigation and N application alter soil microbial communities.

## Materials and Methods

### Site Description and Experiment Design

The study was performed at an experimental field located in Wenxian (34°92′ N, 112°99′E), which is located in the Henan Province of northern China. The experimental site was in a semi-arid area of the Huang-Huai region where crops were grouped in a wheat-summer maize rotation. The annual average temperature in this area is 13.0°C and the annual precipitation is 650 mm; 60–70% of this precipitation occurs in the summer (July–September). Supplementary Figure S1 shows that the precipitation distribution and mean temperature were similar to historical values during the wheat growing seasons in experimental years. The soil is loam (sand 14.1%, silt 47.5%, and clay 38.4%) and the mean soil bulk density is 1.33 g/cm^3^.

In October 2010, treatments were initiated to investigate the effects of irrigation and N application. Yumai 49–198, a high-yield wheat cultivar widely planted in the Huanghuai wheat production area, was used in this experiment. The study design comprised a factorial combination of two irrigation regimes, namely no irrigation (W0) and irrigation at the jointing and booting stages (water sensitive phase of wheat) with 75 mm each time (W2), and two N rates (0 and 240 kg ha^–1^, designated N0 and N2, respectively). Plots were arranged in a split-plot design with four replicates, with main plots assigned to irrigation regimes, and subplots assigned to N rates. Each plot was 2.5 m wide and 6.1 m long. Phosphorus (P) was applied in the form of calcium superphosphate (15%) at a rate of 150 (P_2_O_5_) kg ha^–1^, and potassium (K) was applied using potassium chloride (60%) at 120 (K_2_O) kg ha^–1^ in all treatments. Half of the N (urea, 46% N) and all P and K was spread by hand before ploughing during sowing, while the other half of the N was applied at the jointing stage. A movable sprinkling system was employed watering irrigated plots, and a water meter was used to measure water use.

### Sampling and Analysis

The wheat yield has been recorded each year (2011–2017) of the study. Soil and root samples (plough layer, 0–0.2 m) were analyzed following harvest on June 1 in 2017. Five bulk soil samples were collected by using a soil sampler from each plot and thoroughly mixed to homogeneity as a single sample. Soil samples were obtained four replicates each treatment. Samples were divided into two parts, one of which was air-dried for chemical analysis, and the other was screened through a 2 mm mesh sieve and immediately stored at −80°C for microbial analysis. The soil pH in a saturation extract (1:5 w/v) was measured using a pH meter. Organic matter and total N were measured using the dichromate oxidation method. Available N was determined by the alkaline hydrolysis diffusion procedure, available P was extracted with 0.5M NaHCO_3_ and measured using molybdenum blue spectrophotometry, and available K was determined by displacement with 1 M ammonium acetate followed by flame photometry. The water content of soil samples was determined by oven-drying to a constant weight at 105°C. The content of soil NH_4_^+^ and NO_3_^–^ extracted with 2 M KCl was determined using a continuous flow approach (Santt System, Skalar, Holland). Soil blocks were dug out to obtain root samples. Each sampling area was 0.4 m long (perpendicular to rows, providing access to plants in two rows) and 0.4 m wide (parallel to rows). All samples were placed in a 100-mesh nylon bag, washed with tap water, and clean roots were imaged by gray-scale scanning with an Epson perfection V700 photo instrument. Files were analyzed using WinRHIZO 2008 to determine root length, roots were then dried at 80°C to determine dry weight, and root length density (RLD; m m^–3^) and root weight density (RWD; g m^–3^) were determined from the following formulae:

RLD=L/V

RWD=M/V

where L is the total root length (m), V is the soil sample volume (m^3^), and M is root dry weight (g).

### Soil DNA Extraction, PCR Amplification, and Sequencing

Microbial DNA was extracted from 0.5 g soil samples (fresh weight) using an E.Z.N.A. soil DNA Kit (Omega Bio-Tek, Norcross, GA, United States) according to the manufacturer’s protocols. The final DNA concentration was determined by a NanoDrop 2000 UV-vis spectrophotometer (Thermo Fisher Scientific, Wilmington, United States), and DNA quality was confirmed by 1% agarose gel electrophoresis. The V3 − V4 hypervariable regions of the bacterial 16S rRNA gene were amplified using primers 338F (5′-ACTCCTACGGGAGGCAGCAG-3′) and 806R (5′-GGACTACHVGGGT WTCTAAT-3′) on a thermocycler PCR system (GeneAmp 9700, ABI, United States) (Mori et al., 2013; Xu et al., 2016). Amplification involved denaturation at 95°C for 3 min, followed by 27 cycles at 95°C for 30 s, annealing at 55°C for 30 s, elongation at 72°C for 45 s, a final extension at 72°C for 10 min, and completed reactions were held at 10°C. All reactions were performed in triplicate in 20 μL volumes containing 4 μL of 5× FastPfu Buffer, 0.8 μL of forward primer (5 μM), 2 μL of 2.5 mM dNTPs, and 10 ng of template DNA, and 0.4 μL of FastPfu Polymerase. PCR products were separated on and extracted from a 2% agarose gel, further purified using an AxyPrep DNA Gel Extraction Kit (Axygen Biosciences, Union City, CA, United States), and quantified using QuantiFluor-ST (Promega, United States) according to the manufacturer’s instructions. Purified amplicons were pooled in equimolar concentrations and sequenced on an Illumina MiSeq platform (Illumina, San Diego, CA, United States), yielding 2 × 300 paired-end reads according to standard protocols (sequencing was performed by MajorBio Bio-Pharm Technology Co. Ltd., Shanghai, China). All raw reads have been deposited in the NCBI Sequence Read Archive (SRA) database (Accession Number: SRP217377).

### Quantitative Realtime PCR (q-PCR)

Bacterial abundance was determined by qPCR using primers as described above (Jiang et al., 2019). Ten-fold serial dilutions of plasmid containing the target fragment of the 16S rRNA gene were used to generate a qPCR standard curve, and a Light Cycler 480 instrument (Roche Applied Science, Basel, Switzerland) was used for qPCR with 20 μL reactions containing 10 μL of SYBR Premix Ex Taq, 1.0 μL of 10 μmol L^–1^ forward and reverse primers (each), 7.0 μL of sterilized MiliQ water, and 1 μL of extracted soil DNA. Amplification involved an initial denaturation at 95°C for 30 s (ramp rate = f 4.4°C s^–1^), followed by 30 cycles of denaturation at 95°C for 5 s, annealing and elongation at 60°C for 30 s, and a final cooling cycle at 50°C for 30 s. The bacterial 16S rRNA gene copy number was calculated from the cycle threshold (*C*t) value using a standard curve.

### Processing of Sequencing Data

Illumina MiSeq sequences were processed using QIIME (version 1.70) (Campbell et al., 2010). Raw fastq files were demultiplexed, filtered in terms of quality by Trimmomatic, and merged by FLASH with the following criteria: (i) reads were truncated at all sites with an average quality score <20 over a 50 bp sliding window, (ii) reads containing ambiguous bases were removed and primers were matched exactly allowing two nucleotide mismatches, and (iii) sequences overlapping by more than 10 bp were merged based on overlapping sequence.

Operational taxonomic units (OTUs) were clustered with a 97% similarity cutoff using UPARSE (version 7.1)^1^, and chimeric sequences were identified and removed using UCHIME (Edgar, 2010). Randomly selected sequences from different samples were normalized to the same number of sequences (25,859) among all treatments. The RDP Classifier algorithm^2^ and the Silva (SSU128) 16S rRNA database were employed for assessing the taxonomy of each 16S rRNA gene sequence using a 70% confidence threshold (Pruesse et al., 2007). The most abundant sequence from each OTU was considered representative and taxonomically classified by BLAST searching against GenBank. Bacterial richness and diversity were estimated using OTU richness and Shannon indices, respectively (Chao and Bunge, 2002).

### Statistical Analysis

Data were transformed based on presumed normality and homogeneity of variance where appropriate. Differences between treatments were determined using analysis of variance (ANOVA) followed by least significant difference (LSD) tests. The compositional variations between bacterial communities were visualized using non-metric multidimensional scaling (NMDS) plots based on Bray-Curtis distance in the *Vegan* package. Statistical testing among variation in microbial community composition was carried out using the analysis of similarity (ANOSIM). The ANOSIM was conducted using 999 permutations. In addition to community structure analysis, functional content was inferred by Phylogenetic Investigation of Communities by Reconstruction of Unobserved States (PICRUSt) based on high-throughput 16S rRNA sequencing data, and further analyzed using the Cluster of Orthologous Groups (COG) database (Langille et al., 2013). ANOVA and Spearman’s rank correlation coefficient were used to assess statistical associations among bacterial community, bacterial function, and soil parameters, both within SPSS version 17.0 software.

## Results

### Wheat Yield and Root Growth

The W0N2, W2N0 and W2N2 treatments increased yields by 78.7, 53.1, and 194.6%, respectively, compared with the W0N0 treatment; the N content increased by 47.4, 25.0, and 105.3%, respectively (Table 1). Watering also had a significant impact on root characteristics in the 0−20 cm layer; compared with W0, W2 (average across two N treatments) increased RWD and RLD values by 38.3 and 56.5%, respectively. N application had a clear effect on RWD under the no irrigation condition (W0) and RLD with irrigation (W2). RWD in the W0N2 treatment declined by 22.3% compared with the W0N0 treatment; however, RLD in the W2N2 treatment increased by 15.9% compared with the W2N0 treatment. Significant interactions were also obtained between water and N; this indicates that both wheat yield and root growth in the 0–20 cm are influenced by water, N application, and the combination of the two treatments.

**TABLE 1:** Effects of irrigation and N applied on yield and root characteristics in 0–20 cm.

Treatment	Yield (kg ha^–1^)	RWD (g m^–3^)	RLD (m m^–3^)	N content (%)
W0N0	2942 ± 40d	145.6 ± 3.8b	14826 ± 416c	0.76 ± 0.01d
W0N2	5257 ± 29b	113.2 ± 4.6c	13257 ± 215c	1.12 ± 0.04b
W2N0	4505 ± 4c	175.7 ± 3.1a	20351 ± 1097b	0.95 ± 0.01c
W2N2	8666 ± 106a	182.1 ± 1.6a	23596 ± 1517a	1.56 ± 0.02a
Irrigation (W)	**	**	**	**
Nitrogen (N)	**	**	Ns	**
W × N	**	**	*	**

Numbers after ± are standard errors. Values followed by different letters within each column are significantly different at the probability level of 0.05. ns, not significant, * p < 0.05, ** p < 0.01.

### Selected Soil Properties

Irrigation and N application had effects on soil chemical properties (Table 2). Irrigation increased soil moisture and pH by 9.46 and 2.10% at maturity, respectively (averaged across the N0 and N2 treatments). N application significantly decreased pH under the W0 condition (*P*< 0.05), indicating that soil acidification occurred with W0N2 treatment. N application significantly increased NO_3_^–^N and available N concentrations, but decreased available P concentration. Organic matter and available K concentrations significantly decreased under the W2 conditions.

**TABLE 2:** Properties of soil samples under different fertilizer and water treatments.

Soil properties	W0N0	W0N2	W2N0	W2N2	*P*-value		
					W	N	W × N
Moisture (%)	14.23 ± 0.08b	12.19 ± 0.06d	15.35 ± 0.04a	13.56 ± 0.04c	<0.001	<0.001	0.046
pH	8.32 ± 0.0002b	8.18 ± 0.011c	8.48 ± 0.003a	8.36 ± 0.027b	<0.001	<0.001	0.117
NO_3_^–^N (mg kg^–1^)	11.49 ± 0.43c	28.65 ± 0.35a	6.45 ± 0.04d	24.59 ± 0.26b	<0.001	<0.001	0.131
NH_4_^+^-N (mg kg^–1^)	4.32 ± 0.27b	6.45 ± 0.26a	4.16 ± 0.12b	4.42 ± 0.12b	<0.001	<0.001	0.001
Total N (g kg^–1^)	1.21 ± 0.018a	1.09 ± 0.024b	1.06 ± 0.026b	1.02 ± 0.008c	<0.001	0.078	0.002
Organic matter (g kg^–1^)	14.69 ± 0.045a	13.86 ± 0.054b	13.28 ± 0.041c	13.28 ± 0.065c	<0.001	<0.001	<0.001
Available N (mg kg^–1^)	90.86 ± 0.59c	119.49 ± 0.35b	120.68 ± 1.17b	133.91 ± 1.02a	<0.001	<0.001	<0.001
Available P (mg kg^–1^)	9.13 ± 0.14a	8.71 ± 0.02b	9.12 ± 0.03a	7.88 ± 0.03c	<0.001	<0.001	<0.001
Available K (mg kg^–1^)	160.18 ± 2.57b	185.22 ± 1.30a	137.51 ± 2.46c	136.21 ± 1.47c	<0.001	<0.001	<0.001

*Numbers after ± are standard errors. Different letters in the same row indicate significant difference (P < 0.05) between treatments. Results from two-way ANOVAs with split-plot design were shown in the right panels of the table.*

### Bacterial Abundance and Diversity

**FIGURE 1:**
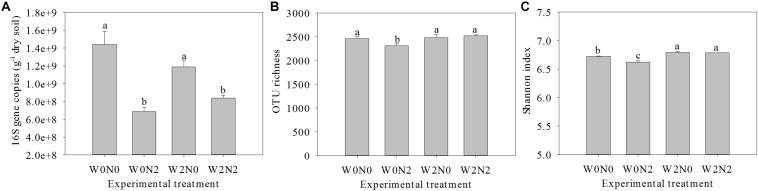
Effects of experimental treatments on bacterial abundance **(A)**, richness **(B)**, and Shannon diversity index **(C)**. Bars with different letters are significantly different (*p* < 0.05).

**FIGURE 2:**
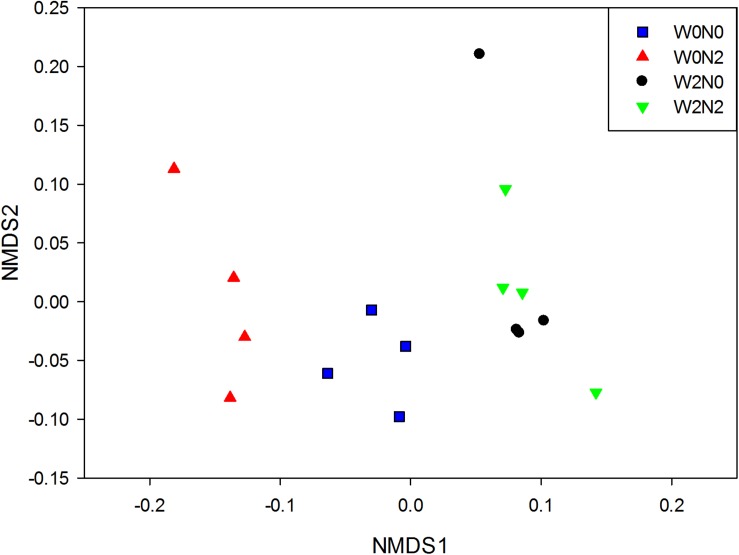
Non-metric multi-dimensional scaling (NMDS) plots of bacterial communities based on Bray-Curtis dissimilarities.

The water and N fertilization regimes influenced the size of the soil bacterial community, based on qPCR analysis of 16S rRNA genes (Figure 1A), and the effect on gene copy number was significant. The number of bacterial 16S rRNA genes in 1 g of soil ranged from 6.8 × 10^8^ to 1.4 × 10^9^, and there was a significant decrease (ANOVA, *p*< 0.05) for N fertilizer treatments. There were 2,461, 2,314, 2,482, and 2,522 OTUs in W0N0, W0N2, W2N0, and W2N2 soils, respectively (Figure 1B). N application decreased richness indices for un-irrigated soils (W0) (*p* < 0.05), but increased these indices for irrigated soils (W2). Compared with the W0 treatment group, the Shannon index in W2 treatment was increased by 1.7% (Figure 1C). Beta-diversity of bacterial communities revealed a significant contribution from irrigation and N application to bacterial community structure variation. Similarity in bacterial communities between samples was compared by Anosim and NMDS based on Bray-Curtis distance (Singh et al., 2015), and the community structure of soil bacteria shifted significantly following water and N addition, with clear distinctions between different treatments (Figure 2; Anosim, *r* = 0.64, *p* = 0.001).

### Responses of Dominant Bacterial Taxa

**FIGURE 3:**
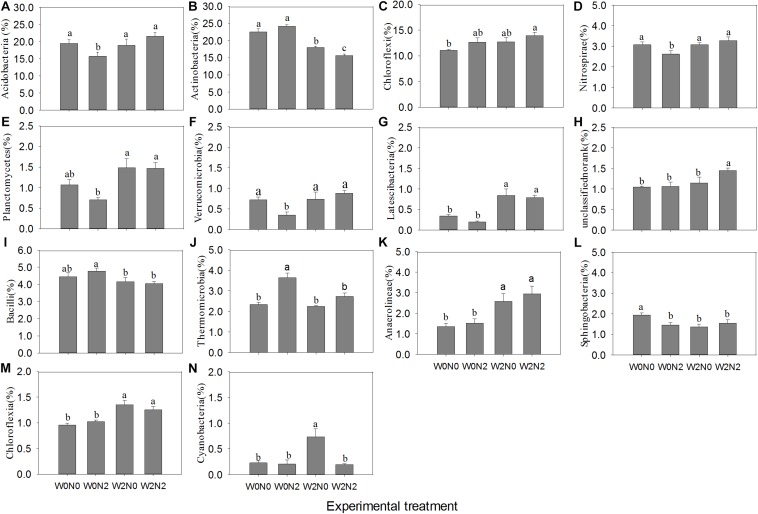
Effects of experimental treatments on the relative abundance of 14 dominant bacterial phyla **(A–H**) and classes **(I–N)**. Only significant statistical results (*p* < 0.05) are shown in the figure for clarity. Bars indicate standard error. Bars with different letters are significantly different (*p* < 0.05).

The most abundant phyla across all treatments were Actinobacteria, Proteobacteria, Chloroflexi, and Acidobacteria. There were lower levels of Nitrospirae, Planctomycetes, Verrucomicrobia, and Latescibacteria (Supplementary Figure S2). The four dominant phyla accounted for 11.09−27.08% of bacterial abundance across all samples. These phyla had different responses to irrigation and N application (Figure 3). Irrigation significantly decreased the relative abundance of Actinobacteria (*p* < 0.05), increased the relative abundance of Planctomycetes and Latescibacteria, and did not significantly alter the relative abundance of Proteobacteria. The relative abundance of Acidobacteria, Nitrospirae, Planctomycetes, and Verrucomicrobia was significantly lower in the W0N2 treatment compared with the other treatments (*p* < 0.05). Irrigation or N application increased the relative abundance of Chloroflexi; the relative abundance of this phyla was significantly higher in the W2N2 treatment than in the W0N0 treatment (*p* < 0.05). There were many alterations at the class level (Supplementary Figure S3). Notably, irrigation significantly increased the relative abundance of Anaerolineae and Chloroflexia while decreasing the relative abundance of Bacilli. Additionally, the relative abundances of Thermomicrobia in the W0N2 treatment and Cyanobacteria in the W2N0 treatment were significantly higher than in the other treatments.

### Functional Prediction of Bacterial Communities

**FIGURE 4:**
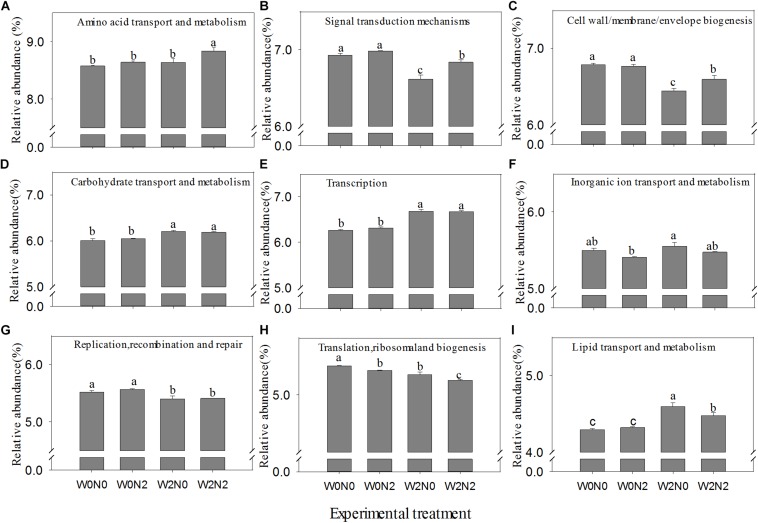
Effects of experimental treatments on the relative abundance of selected function category (main and significant change). **(A)** Amino acid transport and metabolism, **(B)** signal transduction mechanisms, **(C)** cell wall/membrane/envelope biogenesis, **(D)** carbohydrate transport and metabolism, **(E)** transcription, **(F)** inorganic ion transport and metabolism, **(G)** replication, recombination and repair, **(H)** translation, ribosomal structure, and biogenesis, and **(I)** lipid transport and metabolism. Bars with different letters are significantly different (*p* < 0.05).

The relative abundance of functions inferred from PICRUSt analysis is illustrated in Supplementary Figure S4. Compared with taxonomic profiles, the functional profiles of all samples were much more similar to each other. Amino acid and carbohydrate metabolism, general function prediction only, energy production and conversion, and signal transduction mechanisms were the functions most abundant in all samples. Irrigation (W2) treatments had an significant increase in the abundance of carbohydrate transport and metabolism, transcription, inorganic ion transport and metabolism, and lipid transport and metabolism but a decrease in the abundance of signal transduction mechanisms, cell wall/membrane/envelope biogenesis, and replication, recombination and repair (*p* < 0.05). The relative abundance of amino acid transport and metabolism, signal transduction mechanisms, and cell wall/membrane/envelope biogenesis was significantly increased, but translation, ribosomal structure and biogenesis were significantly decreased in N2 treatments (*p* < 0.05; Figure 4).

### Linking Bacteria Communities and Functions to Soil Properties, Wheat Yield, and Root Characteristics

**FIGURE 5:**
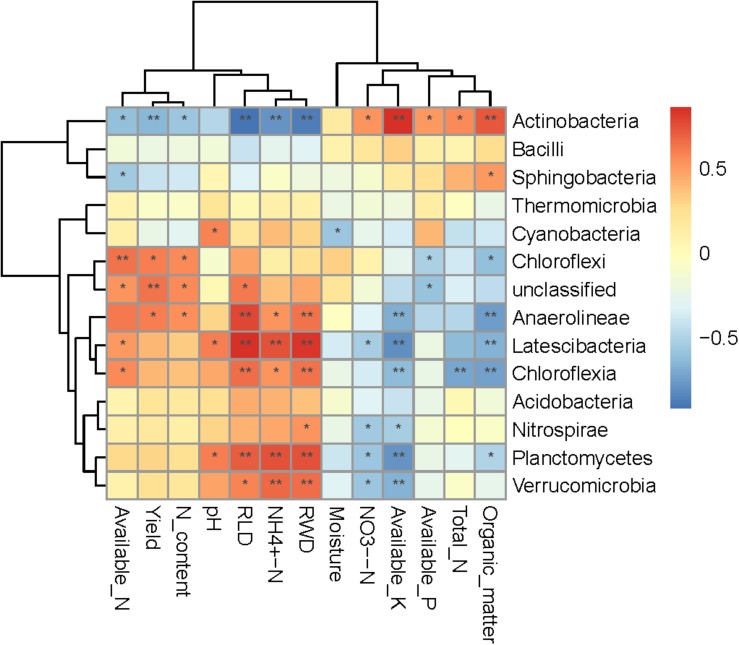
Spearman correlation heatmap between bacterial communities and soil properties and wheat characteristics. *R-*values are displayed in different colors, as indicated by the color code on the right of the heat map.

**FIGURE 6:**
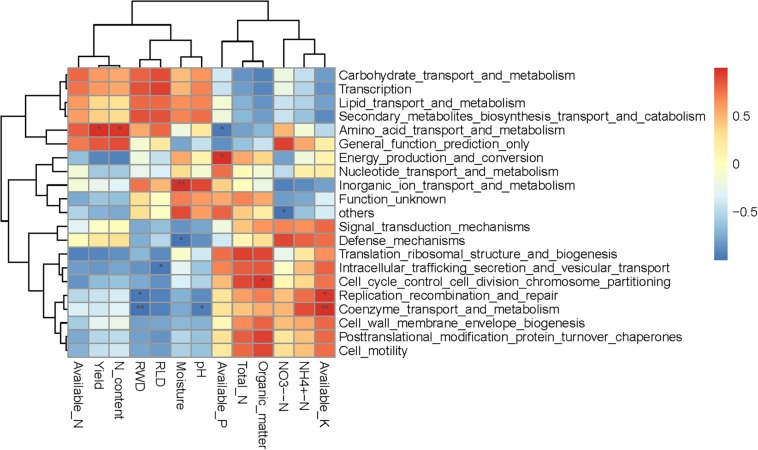
Spearman correlation heatmap between bacterial function and soil properties and wheat characteristics. *R-*values are displayed in different colors, as indicated by the color code on the right of the heat map.

Spearman correlation heatmap analysis was performed to evaluate relationships between environmental factors and select bacterial taxa (both soil and plant; Figure 5). Most bacteria were significantly correlated with at least one environmental factor. The Actinobacteria phylum was significant positively correlated with NH_4_^+^-N, total N, organic matter, available P, and available K, and significant negatively correlated with soil pH, available N, yield, RWD, RLD, and N content. The relative abundances of Planctomycetes and Latescibacteria were positively correlated with soil moisture, pH, RWD, and RLD, and significantly negatively correlated with NH_4_^+^-N and available K. Interestingly, NO_3_^–^-N was only significantly negatively correlated with the Cyanobacteria class. Moreover, available K, RLD, and RWD were significantly correlated with the largest number (seven) of bacterial taxa. We further examined soil and plant variables significantly correlated with the relative abundance of differentially abundant taxa (Table 3). Although the relative abundance of selected groups was significantly correlated with various soil and wheat parameters (Table 3), stepwise regression revealed variation in the relative abundance of select bacterial taxa that was primarily linked to RLD, available N, soil moisture, soil pH, yield, N content, available K, organic matter, and NH_4_^+^-N. In particular, the relative abundance of Actinobacteria was significantly negatively correlated with RLD; meanwhile, the relative abundances of Latescibacteria and Anaerolineae were significantly positively correlated with RLD. Available N was significantly positively correlated with the relative abundance of Chloroflexi but negatively correlated with the relative abundance of Sphingobacteria. Soil moisture was significantly positively correlated with the relative abundance of Chloroflexia and Cyanobacteria. Spearman correlation heatmap between bacterial function and environmental factors revealed that RWD significantly negatively correlated with replication recombination repair and coenzyme transport and metabolism, while available K had opposite effect compared to RWD. Yield and root N content significantly positively correlated with amino acid transport and metabolism. Moisture significantly increased the inorganic ion transport and metabolism but decreased the defense mechanisms (Figure 6).

**TABLE 3:** Variables responsible for the changes in the abundance of various bacterial groups.

Taxonomic group	Model	*R*^2^	*F*	*P*
Actinobacteria	y = 33.936–7.7 × 10^–4^ (RLD)	0.843	75.134	<0.001
Acidobacteria	–			
Chloroflexi	y = 5.235+0.063 (available N)	0.406	9.566	0.008
Nitrospirae	y = 4.19–0.243 (NH_4_^+^)	0.298	5.953	0.029
unclassified	y = 1.868+0.001 (yield)-3.174 (N content)	0.696	14.909	<0.001
Planctomycetes	y = 3.671–0.016 (available K)	0.613	22.197	<0.001
Verrucomicrobia	y = −12.175+1.692 (pH)	0.464	12.095	0.004
Latescibacteria	y = −0.605+6.365 × 10^–5^ (RLD)	0.810	38.509	<0.001
Bacilli	–			
Thermomicrobia	–			
Anaerolineae	y = −0.683+1.54 × 10^–4^ (RLD)	0.599	20.889	<0.001
Sphingobacteria	y = 3.173–0.013 (available N)	0.310	6.295	0.025
Chloroflexia	y = 3.292+0.06 (moisture)-0.217 (OM)	0.656	12.418	0.001
Cyanobacteria	y = −1.52+0.136 (moisture)	0.352	7.598	0.015

## Discussion

### Irrigation and N Application Influence Wheat Yield and Root Characteristics

Irrigation and N application impacts root system growth as well as wheat yield. According to Xue et al. (2003) and Zhang et al. (2004), irrigation frequency is positively correlated with RLD and RWD. We found that irrigation significantly increased both RLD and RWD (47.4% average increase). Previous studies observed that water deficit can increase the number of lateral roots at the soil surface, thereby increasing water absorption (Merrill et al., 2002). However, we showed that irrigation can facilitate root growth. Under no-irrigation conditions (W0), RLD and RWD decreased with N application, indicating that a lower soil water content combined with sufficient N depressed wheat root growth and development (Table 1). Water and N interact to confer complementary effects on root growth and wheat yield (Li et al., 2004), and Zhang et al. (2009) showed that a more developed upper root system is associated with greater shoot biomass, resulting in higher grain yield. In our study, the W2N2 treatment had the highest yield, RLD, RWD, and N content; however, the W0N0 treatment had a higher RLD and RWD than the W0N2 treatment. These results suggest that N application is unable to balance the adverse effect of water stress on the root system under drought conditions, although it does increase grain yield and N content of the root system.

### Effect of Long-Term Irrigation and N Application on Soil Properties

In our study, irrigation increased soil pH from 8.25 to 8.42, which could be because higher soil moisture in the W2 group decreased soil oxygen content and promoted denitrification (Zhang et al., 2014). An intensive microbial nitrification process can lead to soil acidification when large amounts of synthetic N fertilizers are applied, as demonstrated by elevated nitrate levels in soils treated with N (Stamatiadis et al., 1999). Further, N addition can cause soil acidification through the release of free H^+^ by nitrification (Lv et al., 2013; Wang et al., 2016). Zhou et al. (2015) showed that soil pH changed from 6.36 (unfertilized control) to 4.64 after N fertilizer application for 34 years. In our study, there was a relatively small change in soil pH (decreased from 8.32 in the W0N0 treatment to 8.18 in the W0N2 treatment) compared with Zhou et al. (2015); this is likely due to the differences in experiment duration and soil type. Previous studies also demonstrated that long-term N fertilization can increase soil organic C and total N (Jagadamma et al., 2007; Zhao et al., 2016), but these results were different from those of the current study. There were significant differences in NO_3_^–^-N concentration between treatments but not in NH_4_^+^-N levels; these observations agree with previous work by Dong et al. (2014). Accumulation of NO_3_^–^-N in N treatment groups was likely due to the stimulation of nitrification by urea. Furthermore, NH_4_^+^-N from chemical N fertilizer is readily oxidized to NO_3_^–^N in dryland soils.

### Irrigation and N Application Impact Soil Bacterial Diversity and Microbial Community Composition

N application significant decreased the bacterial richness and diversity under the no irrigation condition; however, this effect is reversed with irrigation (Figure 1). Previous studies revealed consistent effects of water and N on soil bacterial diversity (Ramirez et al., 2010; Zhang et al., 2014). Herein, we showed that N application significantly decreased the relative abundance of Verrucomicrobia, Acidobacteria, and members of the nitrifying community (i.e., Nitrospirae) under no-irrigation conditions (Figure 3), consistent with previous observations (Ramirez et al., 2010; Zhou et al., 2015). Fierer et al. (2012) considered Acidobacteria to be an oligotrophic taxon with a slower growth rate and ability to maintain metabolic activity in nutrient-poor conditions. This demonstrates that available nutrients are utilized by copiotrophic taxa and have a negative effect on oligotrophic taxa. However, Bates et al. (2011) and Zhang et al. (2014) reported that the relative abundance of Nitrospirae increased with N fertilization. Coincidentally, our results show that N application increased the relative abundance of Acidobacteria, Verrucomicrobia, and Nitrospirae under the irrigation condition (Figures 3A,D,F). The relative abundance of Chloroflexi increased under N addition conditions in the present work (Figure 3C), similar previous studies (Zeng et al., 2016; Yu et al., 2019). However, Will et al. (2010) reported that Chloroflexi was increased in nutrient poor soil layers, and a negative correlation with nitrate concentration (Fierer et al., 2012). The mechanism underpinning this reduction in abundance with increasing N fertilizer remains unclear (Wang et al., 2018). Based on these results, it is necessary to explore how N application alters the bacterial community composition. One possible explanation is through changes in soil chemical properties, such as pH and water content. Alternatively, bacteria in oligotrophic phyla may be inhibited by other bacterial groups that increase in abundance following N application through nutritional effects and/or the production of toxic products (Eo and Park, 2016). Further research is needed to determine exactly how biotic and abiotic factors influence bacterial community composition.

Moreover, changes in soil bacterial diversity may not be correlated with community function (Lupatini et al., 2013). For example, Pan et al. (2014) suggested that long-term inorganic fertilization does not affect microbial function, since functional redundancy maintains ecosystem function if bacterial composition is altered. Elucidating the functional roles of dominant bacterial groups influenced by irrigation and N application will require further work.

### Irrigation and N Application Impact Soil Bacterial Functions

Our results clearly showed that N fertilizer and irrigation treatment influenced bacterial community structure. However, their effects on wheat soil bacterial community and associated 16S rRNA gene-predicted functions under field conditions remain poorly understood (Kavamura et al., 2018). In general, copiotrophs are enriched in COGs related to motility, defense mechanisms, transcription, and signal transduction. In contrast with copiotrophs, oligotrophs are enriched in COGs associated with lipid transport and metabolism, secondary metabolite biosynthesis, and transport and catabolism (Lauro et al., 2009). In our results, the N fertilizer and irrigation increased the relative abundance of signal transduction mechanisms and transcription, respectively (Figure 4). However, lipid transport and metabolism enriched in N0 treatments. It is possible that oligotrophs preferentially use lipids for carbon and energy both directly and for storage (Lauro et al., 2009). Our results are consistent with those of Fierer et al. (2012) and Kavamura et al. (2018), who showed that soil samples treated with either medium or high levels of inorganic N were associated with predicted functions related to amino acid metabolism, suggesting that wheat secretes large amounts of amino acids through roots when sufficient N is present, and amino acid metabolism is promoted in soil bacteria. However, Wang et al. (2018) reported that N0 treatment enriched the functions related to amino acid transport and metabolism. One possible reason is the difference of pH range (5.10 in Wang et al. and 8.18∼8.48 in our studies, respectively) in N treatment. Wang J. et al. (2014) stated that irrigation increased the relative abundance of carbohydrate transport and metabolism by promoting the plant growth, and this conclusion is also supported by our current results.

### Mechanisms by Which N Application and Irrigation Alter Bacterial Community Structure and Function

We found that soil and wheat characteristics played important roles in driving bacterial community composition. Previous findings suggest that at the continental scale, pH is a universal predictor of bacterial composition (Lauber et al., 2009). Zhao J. et al. (2014) described that the Acidobacteria and Chloroflexi were sensitive to soil pH and Actinobacteria was poorly correlated with soil pH. Although pH was not significantly correlated with the relative abundance of Chloroflexi and Acidobacteria, it was significantly negatively correlated with Actinobacteria (Figure 5). The discrepancies with previous studies may be due to the fact that our study was conducted over a smaller pH range than previous studies, where the soil pH varied from 3 to 8. We found several soil properties that were significantly correlated with the relative abundance of bacterial taxa, including soil moisture content, available P, available N, available K, and organic matter (Table 3). Actinobacteria are copiotrophic (thrive under elevated C and N conditions and exhibit relatively rapid growth rates), can be both aerophilic or microaerophilic, and are involved in organic matter degradation in soils (Eilers et al., 2010; Zhou et al., 2015). We found that the relative abundance of Actinobacteria was positively correlated with organic matter, available P, and available K, and negatively correlated with available N (Figure 5). The relative abundance of Verrucomicrobia and Chloroflexi was negatively correlated with soil nutrients (total N, organic matter, available P, and available K) in our study; this finding agrees with those of a previous study on prairie soils (Fierer et al., 2013) because Verrucomicrobia are generally considered to be oligotrophic (Zhalnina et al., 2015). Overall, taxa abundance was influenced by physicochemical factors (Table 3). Plant factors were also correlated with bacterial abundance. There are limited data available regarding the correlations between crop yield and specific microbes (Zhou et al., 2015). Li et al. (2016) reported that plant biomass was significantly positively correlated with the relative abundance of Proteobacteria, TM7, OD1, and Gemmatimonadetes, and significantly negatively correlated with the relative abundance of Acidobacteria and Chloroflexi. Our results show that taxa abundance is closely related to wheat factors. Yield, RWD, RLD, and N content were significantly negatively correlated with the relative abundance of Actinobacteria and positively correlated with most other bacterial taxa. Using stepwise regression, we found that RLD was most significantly correlated with the relative abundance of bacterial taxa (Figure 5). This is likely due to the large amount of root residues in the soil and the increased release of root exudates, which can impact soil bacterial abundance.

In bacteria, genes encoding terpene synthases are mostly related to the production of secondary metabolites under limited N conditions (Yamada et al., 2015). In copiotrophs, the proportion of extracytoplasmic proteins (cytoplasmic membrane, periplasmic, outer membrane, and extracellular) is higher than in other organisms (Lauro et al., 2009). In the present study, we obtained similar results; a nutritious environment (higher available K, NO_3_^–^N, NH_4_^+^-N, and organic matter) suppressed secondary metabolite biosynthesis, transport and catabolism, but promoted cell wall membrane envelope biogenesis. In the present study, the relative abundance of taxa and genes was assessed based on 16S rRNA gene amplicon sequencing and PICRUSt, respectively. However, the results should be compared with metagenomics and metatranscriptiomics in future experiments because of the limitation of PICRUSt function prediction.

## Conclusion

This study demonstrates that long-term irrigation and N application significantly influences wheat plant characteristic, soil properties, and bacterial community composition. N fertilizer led to soil acidification and decreased the abundance, richness and diversity under the no irrigation conditions. However, irrigation partly buffered the effect of N application, which suggests that concurrent irrigation and N application can improve wheat yield and maintain microbial ecosystem function. The results solidify the relationship between bacterial taxa and soil properties; we also found that some bacterial taxa were significantly correlated with plant properties, specifically root characteristics. Additionally, the treatments also changed some potential functions of bacteria by influencing the soil and plant characteristics. Overall, we provide insights into the effects of N application and water management strategies with the goal of improving agricultural practices. Future studies should focus on the influence of agricultural management practices on rhizosphere soil microbial function using metagenomics and metatranscriptomics, and their relationships with the plant.

## Data Availability Statement

The BioProject ID is PRJNA558920, accession number is SRP217377. You can find the data here: https://www.ncbi.nlm.nih.gov/sra/?term=SRP217377.

## Author Contributions

CW conceived the research and designed the study. GM analyzed the data and wrote the manuscript. GM, JK, JW, and YC carried out the field measurements and soil analysis. HL, LW, and YX critically reviewed the manuscript. DM and GK assisted with manuscript writing and editing. All authors approved the final version of the manuscript.

## Conflict of Interest

The authors declare that the research was conducted in the absence of any commercial or financial relationships that could be construed as a potential conflict of interest.

## Footnotes

1 http://drive5.com/uparse/

2 http://rdp.cme.msu.edu/

## Supplementary Material

FIGURE S1 Monthly precipitation and mean temperature during winter wheat growing season from 2011 to 2017 and the corresponding average from 1962 to 2010 at the experimental site.

FIGURE S2 Mean relative abundances of dominant Phylum-level group in soils treated with Nitrogen and Water.

FIGURE S3 Mean relative abundances of dominant Class-level group in soils treated with Nitrogen and Water.

FIGURE S4 Mean relative abundances of function predicted in soils treated with Nitrogen and Water.
